# International competencies of nurses with advanced practice in anesthesia nursing: An integrative review

**DOI:** 10.1016/j.ijnsa.2025.100319

**Published:** 2025-03-17

**Authors:** Kathrin Julia Pann, Verena-Katrin Buchner, Roland Eßl-Maurer, Tobias Bacher, Manela Glarcher, Andre Ewers

**Affiliations:** a Nursing Department, Coordination of Clinical Nursing Science and Research, University Hospital Salzburg, Paracelsus Medical University, Salzburg, Austria; b Nursing Department, Coordination Development Clinical Nursing Practice, University Hospital Salzburg, Paracelsus Medical University, Salzburg, Austria; c Institute of Nursing Science and Practice, Paracelsus Medical University, Salzburg, Austria; d Department of Anesthesiology at University Hospital Salzburg, Paracelsus Medical University, Salzburg, Austria

**Keywords:** Advanced practice nursing, Advanced practice registered nurses, Anesthesia nursing, Clinical competence, Literature review, Nurse anesthetists, Scope of practice

## Abstract

**Background:**

Internationally, nurse anesthetists play a pivotal role in the highly specialized field of anesthesia care, which requires advanced knowledge. In Austria, advanced nursing practice in anesthesia care lacks a model that allows for an expanded role. Thus, there is a need to explore the scope of practice, advanced competencies, and skills of nurse anesthetists to address the challenges in the provision of healthcare.

**Objective:**

The aim of this review was to explore the competencies and skills of advanced practice nurses in anesthesia care at the international level.

**Design:**

An integrative literature review was conducted using Whittemore and Knafl's five-step methodology.

**Methods:**

A systematic literature search was performed in the databases PubMed, the Cumulative Index to Nursing and Allied Health Literature, the Cochrane Library, and Web of Science. Keywords such as ’nurse anesthetist’, ’nurse practitioner’, ’advanced nursing’, ’advanced practice’, ’professional competence’, ’role ’, ’clinical competence’ and related terms in American and British spelling were used. In addition, references in the included publications, databases for doctoral theses, and non-listed international journals on anesthesia care, as well as studies from websites of professional societies were searched. The quality of included publications was assessed using critical appraisal tools and a qualitative content analysis approach was used to synthesize the data.

**Results:**

In total, 26 publications from 13 different countries were included. Eight qualitative, ten quantitative, and one mixed methods study, as well as seven scientific reports could be identified. The seven main themes - professional competencies, communication competencies, coordination and management competencies, scholarship competencies, advocacy competencies, collaboration competencies, and clinical competencies - could be extracted from the literature. These were supplemented by eleven inductive sub-themes.

**Conclusions:**

Nurse anesthetists demonstrate a comprehensive set of competencies that encompass technical as well as non-technical skills, enabling them to provide high-quality and advanced anesthesia care. There are variations in the scope of practice, roles, and skills of nurse anesthetists across different countries. In some countries, nurse anesthetists practice within a broad scope, while in other countries, often due to policy considerations, their responsibilities and skills are limited.

**Registration:**

PROSPERO CRD42022351430, registered 15/08/2022

**Social media abstract:**

Nurse anesthetists' scope of practice varies internationally due to policies but covers a comprehensive range of competencies @GlarcherManela @KathrinPann

**What is already known**

• In some countries, nurse anesthetists provide advanced anesthesia care.
• In Austria, nurse anesthetists' roles and advanced competencies are undefined.

Alt-text: Unlabelled box

**What this paper adds**

• The nurse anesthetist role is still evolving in certain countries.
• Variations in the scope of practice of nurse anesthetists could be identified.
• This review outlines nurse anesthetists' competencies and skills internationally.

Alt-text: Unlabelled box

## 1 Background

The rising prevalence of multimorbidity and chronic diseases, coupled with changes in hospital policies towards a reduction in inpatient length of stay as well as medical-technical advancements, have led to a higher complexity in nursing care (Aiken et al., 2017). Particularly in the field of highly specialized inpatient care, an increase in complexity and patient acuity is described (Moffat and Mercer, 2015). The operating room is a highly dynamic, complex, and task-intensive setting. To manage expected as well as unexpected events, advanced knowledge, competencies, and skills are required from nurse anesthetists and other healthcare professionals involved (Göras et al., 2020).

Internationally, advanced practice nurses, with various specializations and competencies beyond the level of a generalist nurse, address a wide range of healthcare challenges and can thus meet the changing conditions in healthcare systems (International Council of Nurses, 2020). According to the International Council of Nurses (2020) *“an Advanced Practice Nurse is a generalist or specialised nurse who has acquired, through additional graduate education (minimum of a master's degree), the expert knowledge base, complex decision-making skills and clinical competencies for Advanced Nursing Practice, the characteristics of which are shaped by the context in which they are credentialed to practice”*(p.6). Nurse anesthetists are classified as advanced practice nurses in anesthesia care with a minimum post-graduate education level of a master's degree (International Council of Nurses, 2021).

Unlike countries such as the US, where the nurse anesthetist forms part of the four primary advanced practice nursing roles (Tracy and O'Grady, 2019), in Austria, the development of advanced nursing practice is still evolving. However, it faces challenges, such as the absence of legal guidelines regarding competencies (Austrian Association of Advanced Nursing Practice, 2022), lack of job descriptions, title protection, and role development based on organizational needs (Glarcher & Lex, 2020). At present, Austrian law does not outline educational prerequisites nor does it define an expanded scope for advanced practice nurses in general, including nurse anesthetists (Them et al., 2018). The "Scope of Practice" refers to the range of services that licensed professionals are considered capable of performing and authorized to carry out in accordance with the conditions outlined in their professional license (National Library of Medicine, 2020). The International Federation of Nurse Anesthetists (2016) has defined standards of practice, roles, as well as competencies for nurse anesthetists and developed a framework for anesthesia practice. The framework, based on the “Canadian Medical Education Directives for Specialists” model (CanMEDS), comprises of seven interacting roles that establish them as experts in their field of practice. Nurse Anesthetists are Experts, Communicators, Collaborators, Managers, Health Advocates, Scholars, and Professionals. The International Council of Nurses (2021) characterizes each role based on educational preparation, the nature of practice, and regulatory mechanisms. However, to account for differences in educational systems and policies among different countries, a loophole was created by stating that the distinct attributes of advanced practice are influenced by the individual country and regional settings where nurses are authorized to practice (International Council of Nurses, 2020). The International Federation of Nurse Anaestethists (2024) strives to promote educational standards and recognizes educational programs. However, they have accredited only 13 programs so far, seven of these in US universities.

In 2016, the Austrian nursing education system underwent changes, with nurses obtaining a bachelor's degree upon the completion of their generalist nursing education at universities of applied sciences (Health and Nursing Act, 1997, §1, Education Regulation). As a result, the former specialization course in anesthesia care was transferred to the European Credit Transfer System. Austrian nurses practicing in anesthesia care are required to complete specialization training with a duration of one year and 60 European Credit Transfer System Points. Completion of this specialization allows nurses to hold the title “Academic Expert” (Health and Nursing Act, 1997, §70a; Health and Nursing Act, 1997, Special Regulation 452; Paracelsus Medical University, 2025). Thus, Austrian nurses working in the field of anesthesia can be classified as anesthetic nurses (Schwaiger and Müller, 2022) and support anesthesiologists in the provision of anesthesia (Health and Nursing Act, 1997, §20). On the contrary, in countries such as the US, Sweden, France, and Switzerland, the nurse anesthetist role, in the sense of *“the expected function of a member of the nursing profession”* (National Library of Medicine, 2002), is well established and allows practice with a higher degree of autonomy, including the administration of anesthesia to “American Society of Anesthesiologists” I and II patients (Schwaiger and Müller, 2022). American Society of Anesthesiologists I refers to healthy patients and II to patients with a mild disease (American Society of Anesthesiologists, 2020).

In Europe, there is a distinction between nurse anesthetists and anesthetic nurses. Depending on the country's policies nurse anesthetists may perform anesthetic procedures under direct or indirect supervision, whereas anesthetic nurses play an additive role in assisting anesthesiologists during and after procedures (Schwaiger and Müller, 2022). Conversely, in some US states, nurse anesthetists practice as independent anesthesia providers, while in other states, their scope of practice is limited (Egger Halbeis and Schubert, 2008).

The establishment of the nurse anesthetist role as an advanced practice provider within the Austrian healthcare system, along with the development of a specialized post-graduate curriculum, may present a viable solution to meet the increased demands in this complex field of work. At present, there is no possibility for Austrian anesthetic nurses to expand their competencies and scope of practice through postgraduate study at the master's level. Given the shortage of anesthesiologists in Austria (Markstaller, 2019), exploring the potential of expanding the scope of practice of anesthetic nurses towards an advanced nursing role could be beneficial. Moreover, other countries wherein the nurse anesthetist role has not yet been established, may gain additional insights into the advanced competencies of nurse anesthetists, especially as the International Council of Nurses (2021) describes a need for anesthesiologists and nurses specialized in anesthesia in some low- and middle-income countries.

### 1.1 Aim

This integrative review aimed to explore the current state of competencies and skills of advanced practice nurses in anesthesia care on an international level. The findings of the included studies may serve as a basis for the development of competencies for advanced nursing practice in anesthesia care in Austria.

The following research questions guided the review process:

Which competencies of advanced practice nurses in anesthesia care are described in international literature?

Which skills of advanced practice nurses in anesthesia care are described in international literature?

## 2 Methods

### 2.1 Review design

To present the current global status of competencies of advanced practice nurses in anesthesia care, an integrative review was conducted using the methodology of Whittemore and Knafl (2005), consisting of the five steps of problem identification, literature search, data evaluation, data analysis, and presentation. Integrative reviews in nursing are an appropriate approach for compiling a comprehensive corpus of evidence from diverse methodologies (Whittemore and Knafl, 2005); therefore, this review approach was selected.

### 2.2 Literature search

The junior researcher (K.P.) conducted a systematic literature search in PubMed, the Cumulative Index to Nursing and Allied Health Literature (CINAHL), the Cochrane Library, and Web of Science on August 12, 2022. The search strings were developed in collaboration with experts in the development of advanced nursing practice roles, the professional expertise of the university librarian, the Austrian Association of Advanced Nursing Practice, and the Akademische Fachgesellschaft international. To ensure literature saturation, a hand search of the references in the included publications, databases for doctoral theses, and non-listed international journals on anesthesia care, as well as studies from websites of professional societies was conducted. Only publications involving human participants, regardless of age or cultural background, and published between 2007 and 2022 were included. The review was limited to publications in English and German language focusing on acute care settings (hospital/acute care/inpatient stay). Publications that exclusively referred to opinions of individual experts and political discussions were excluded. Search strings were constructed separately for each database based on the “Population, Phenomenon of Interest, Context” scheme and included – among others – the following search terms: “nurse anesthetist”, “anesthesia nurse”, “certified registered nurse anesthetist”, “nurse practitioner”, “advanced nursing”, “advanced practice”, “professional competence”, “role”, “clinical competence”, “skills”, “anesthesia department”, “recovery room”; “emergency service”. The full PubMed search string is displayed in supplementary material 1.

**Fig. 1:**
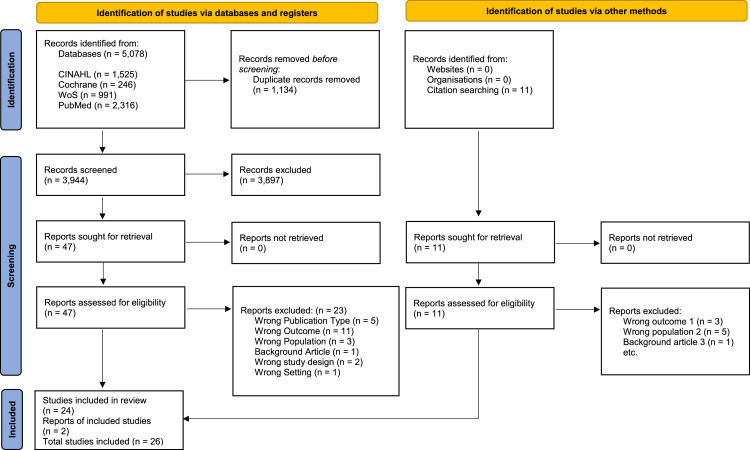
Prisma 2020 flow diagram.

In total the search yielded 5078 records (CINAHL n= 1525, Cochrane Library n= 246, WoS n= 991, PubMed n= 2316). After 1134 duplicates were removed, 3944 titles and abstracts were screened by two researchers with the support of the software Rayyan© (K.P., T.B). Ultimately, 47 full-text articles from databases and 11 articles from additional sources were assessed for eligibility, resulting in a total of 26 publications included in the review. The screening process was documented according to the Preferred Reporting Items for Systematic Reviews and Meta-Analyses (PRISMA, 2020) flow diagram (Fig. 1).

### 2.3 Data evaluation and quality appraisal

Two researches (K.P., T.B.) appraised the quality of the 26 remaining articles using the Joanna Briggs Critical Appraisal Tools (2020) for analytical cross-sectional studies, text and opinion, prevalence studies, and qualitative research. Mixed Methods studies were analyzed with the Mixed Methods Appraisal Tool (Hong et al., 2018). The evidence level was determined by the Johns Hopkins Evidence Level and Quality Guide (Dang et al., 2022) (supplementary material 2). A third researcher (R.E.M) resolved disagreements.

### 2.4 Data analysis

Data extraction was performed in tabular form by one researcher (K.P.) and reviewed by the whole research team. The study characteristics are displayed in Table 1.

**Table 1:** Study characteristics.

Reference, country	Purpose	Methods	Sample	Key findings
Abelsson et al. (2021) Empowerment in the perioperative Dialog Nursing Open Sweden	Description of how the nurse anesthetist empowers the patient in the perioperative dialogue	Qualitative descriptive design Hermeneutic text interpretation	12 nurse anesthetists (NA)	Findings were presented along Gibsons's Empowerment Model (1991): Helper, Supporter, Counsellor, Educator, Consultant, Resource mobilizer, Facilitator The relationship between nurse anesthetists and patients evolves through closeness
Averlid and Høglund (2020) Operating room as a learning arena NA and student perceptions Journal of Clinical Nursing Norway	Identification of mentoring core competencies	Qualitative study Focus groups	8 students 7 NAs	Two main categories and four subcategories: Mentoring in the operating room Creating a good climate for learning
Callan et al. (2021) Impact of COVID-19 Pandemic on CRNA Practice AANA Journal USA	Description and quantification of practice changes as a result of federal regulatory modifications in response to the pandemic	The quantitative part of a mixed methods design Survey	44,100 certified registered nurse anesthetists were eligible 2097 participants were included as they practiced during the first months of the pandemic	84 % practiced in their original role as CRNA 16 % reported an expansion in their scope of practice due to COVID, mainly outside the operating room 16 % stated they worked in COVID Team, 9,5 % COVID inpatient unit, Expanded tasks performed: tracheal intubation (n= 129), ventilator management (n= 82), arterial line placement (n= 76), central line placement (n= 49) Likelihood for expanded practice was higher in states with supervision (major impact state) OR: 1.83 (*p* = 0.001), no sig. effect for opt out states
Chen et al. (2020) Role of Anaesthesia Nurses in Treatment and Management of COVID-19 Patients Journal of perianesthesia nursing China	Discussion of the specialties and roles of anesthesia nurses in front-line COVID-19 work.	Article	n.a.	Description of the scope of practice of Chinese anesthesia nurses Anesthesia nurses have superior competences, which are advantageous for the care of COVID-19 patients. Anesthesia nurses in China mainly work in PACUs and provide postoperative care 27 of the 34 anesthesia nurses of the Union Hospital in Wuhan provided care for COVID-19 Patients for example in fever clinics, isolation wards, ICUs, and cabin hospitals
Dahlberg et al. (2022a) Levels of education and technical skills in RNs working in the PACU Scandinavian Journal of Caring Sciences Sweden	Description and comparison of educational levels and technical skills of RNs working in PACUs Exploration of the desired knowledge of PACU nurses according to nurse managers	Descriptive cross-sectional study	Nurse managers n= 51 Response rate 58 %	**Education:** In 53 % of the PACUs 80 % of the RNs were specialized nurses (mainly intensive care), In 41 % of PACUs 1–79 % of RNs were specialist nurses, In 6 % of the PACUs 100 % of Nurses were without a postgraduate diploma **Skills** High level of autonomy in RNs however significant differences in autonomy between RNs with postgraduate diplomas vs RNs without a postgraduate diploma Postgraduate diploma specialist nurses acted more autonomously compared to RNs
Dahlberg et al. (2022b) Nurse competence in the post-anaesthesia care unit in Sweden: a qualitative study of the nurse's perspective BMC Nursing Sweden	1 Exploration and description of nurses’ perception of the competence needed to work in a PACU 2 Exploration and description of the characteristics of an expert nurse in the PACU	Qualitative inductive study Individual interviews Thematic analysis	PACU nurses n= 16 Convenience sample	Three main themes and six sub-themes were identified: “Being adaptable in an ever-changing environment” “creating safe care” “seeing the bigger picture”
Dahlberg et al. (2021) Education, Competence, and Role of the Nurse Working in the PACU: An International Survey. Journal of PeriAnesthesia Nursing Sweden	Description of roles, education, and competence of nurses working in the PACU in 11 countries.	A descriptive international cross-sectional study	Experts of members countries of the International Collaboration of Peri-Anesthesia Nurses n= 11	In six out of 11 countries perianesthesia nursing is a specialty 8 countries have national guidelines or standards of practice 3 countries have a formal education program
Egger Halbeis and Schubert (2008) Staffing the operating room suite: perspectives from Europe and North America on the role of different anesthesia personnel Anesthesiology Clin Europe & North America USA	n.a.	Article	n.a.	In the US CRNAs replace anesthetists in some areas, or work under supervision In the opt-out states (14) of the US some CRNAs practice independently without medical direction or supervision In contrast no European country allows CRNAs to practice without supervision, variations in job titles, education, degree, scope of practice are identified
Everson et al. (2021) From the Operating Room to the Front Lines: Shared Experiences of Nurse Anaesthetists During the Coronavirus Pandemic AANA Journal USA	Assessment of the impact of the COVID-19 pandemic on CRNA practice	Qualitative component of a Mixed Methods Study	n= 29 CRNAs Purposive and snowball sampling	6 Themes: CRNAs as part of the solution Doing whatever it takes CRNAs are valued contributors Removal of barriers promotes positive change Trying times Expertise revealed
Federico (2007) Innovations in care: the nurse practitioner in the PACU Journal of perianesthesia nursing USA	The project aims to provide continuity of care and increase the availability of post anesthesia care, using an independent practitioner.	Short communication	n.a.	The article describes the privileges and practice patterns of the future PACU nurse practitioner in an urban medical center **Delineation of privileges** diagnostic tests, prescribing, treatments and general care …. **Daily practice patterns** *Direct care* *Indirect care*
Fynes et al. (2014) Anaesthetic nurse specialist role: leading and facilitation in clinical practice Journal of perioperative practice Northern Ireland, UK	To demonstrate the anesthetic nurse specialist role and their leadership role in clinical practice	Discussion	n.a.	Differentiation between transactional and transformational leadership Dynamic situation in the OR requires quick adaptions of leadership style, therefore knowledge of and skills in leadership are important for CRNAs
Halakou et al. (2017) The Clinical Competencies of Nurse Anesthetists in Response to Community Needs: A Delphi Study Journal of Clinical and Basic Research Iran	Determination of the clinical competencies of NAs for meeting the needs of the community.	Delphi study	Stage 1 n= 19 Stage 2 n= 41 Stage 3 n= 39	Agreement on 34 items
Herion et al. (2019) Validating international CanMEDS-based standards defining education and safe practice of nurse anesthetists International Nursing Review Switzerland	Determination if CanMEDS International Federation of Nurse Anesthetists (IFNA) standards define Swiss NAs scope of practice	Cross sectional study Survey: 76 competencies of IFNA standards of practice	n= 449 (response rate 61 %)	All seven IFNA roles were ranked relevant or very relevant *(mean = 4.22, standard deviation SD = 0.42)* The participants rated 82 % of the graduate competencies as relevant or very relevant (m =4.45, SD 0.71) Internal consistency total: 0.97 Factor analysis: The exploratory factor analysis yielded seven factors which accounted for 72 % of the variance, loadings from 0.4 to 0.84
Ide et al. (2020) Introduction of evolving roles of Japanese perianaesthesia nurses Journal of Anesthesia Japan	Introduction of perianesthesia nurses (PAN)	Special article	n.a.	PANs work as advanced practice nurses according to facility policies PANs care for American Society of Anesthesiologists (ASA) 1 and 2 patients Scope of practice e.g. insert intravenous lines intubate patients with video laryngoscopy under direct supervision assist anesthesiologists with ASA 3 or more patients
Lauridsen et al. (2015) Organisation of in-hospital cardiac arrest teams – a nationwide study Resuscitation Denmark	Description of the composition of in-hospital cardiac arrest teams and review pre-arrest allocation of tasks	Cross-sectional study Data from hospital protocols	Cardiac arrest teams descriptions n= 44	All teams included a nurse anesthetist In 76 % airway handling is performed by nurse anesthetists The administration of medication is in 38 % carried out by nurses and in 8 % by nurse anesthetists and anesthesiologists
Matsusaki and Sakai (2011) The role of Certified Registered Nurse Anesthetists in the United States J Anesth USA	Description of the CRNAs role in the US	Short communication	n.a.	Each state can decide for or against the need for CRNAs supervision In 16 states, there is no obligation for CRNAs to be supervised by anesthesiologists, surgeons or dentists. In sates with mandatory supervision an anesthesiologist supervises two to four CRNAs and has to be present during defined anesthesia phases Article describes CRNAs scope of practice
Meeusen et al. (2010) Composition of the anaesthesia team: a European survey. European Journal of Anaesthesiology Europe	Assessment of availability, roles and functions of non-physician anesthesia team members in European countries	Quantitative descriptive cross sectional study	n= 31 European countries	Each European country has different roles and responsibilities of non-physician anesthesia providers Scope of practice and education of European nurse anesthetists should be standardized
Neft et al. (2013) Revised Scope of Practice Embodies the Broad Continuum of Nurse Anesthesia Services AANA Journal USA	Gain understanding of the full scope of nurse anesthesia practice	Systematic Literature Review 6 Focus Groups: CRNAs and students Survey	Focus Groups n= 55 Survey 4200 CRNAs (response rate 14.5 %)	**Focus Group Themes:** Current elements of nurse anesthesia practice in education programs, rural community and university medical centers, Future practice opportunities Inter-professional collaboration Autonomous practice Barriers to practice and recommendations **Survey** 45 % reported that they are not allowed to practice the full scope of practice, Restrictions in chronic pain management (77 %), transoesophageal echo probe insertion (71 %), peripheral nerve catheter placement (70 %)
Olin et al. (2022) Mapping registered nurse anaesthetists’ intraoperative work: tasks, multitasking, interruptions and their causes, and interactions: a prospective observational study. causes BMJ Open Sweden	Mapping registered nurse anesthetists’ work including tasks, multitasking, interruptions, causes, interactions in all phases of the work process.	Prospective observational study Structured observations	n= 8 registered nurse anesthetists during 30 procedures total of 73h of observations One hospital	High intensity of tasks and multitasking during: preparation for anesthesia induction (79 tasks/hour, 61.9 % of task time multitasking) anesthesia induction (98 tasks/h, 50.7 % multitasking), preparation for anesthesia maintenance (86 tasks/hour, 80.2 % multitasking). CRNAs worked mostly independently (58.4 %) Highest proportion of time was spent on indirect care (52 hours 55 min, 41.9 %), supervision (23 hours 11 min, 18.4 %) and direct care (12 hours 16 min, 11.7 %).
Rayborn et al. (2017) The Future of Certified Registered Nurse Anesthetist Practice in South Korea: Fading Into the Sunset or Breaking of a New Dawn AANA Journal South Korea	Description of practice of nurse anesthesia in Republic of Korea. Identification if trends, policy and other factors affect the profession.	Cross sectional survey study	267 surveys analyzed anesthesia registered nurses n= 141, CRNAs n= 126	Significant differences in practice and job satisfaction related to level of education Significant differences in practice between CRNA and anesthesia registered nurses
Rönnberg et al. (2019) The Art is to Extubate, Not to Intubate – Swedish Registered Nurse Anesthetists’ Experiences of the Process of Extubation After General Anesthesia Journal of Perianesthesia Nursing Sweden	Description of Registered Nurse Anesthetists’ experiences of the process of extubation in patients undergoing general anesthesia	Qualitative study Focus groups (n= 3) Content analysis	n= 20 registered nurse anesthetists consecutive sampling strategy Setting: University Hospital 9 country hospitals	Four categories and eight subcategories emerged Main categories: To be a step ahead To be on my Toes To use Situation Awareness To be Alone in a Critical Moment The decision for the timing of extubation is made based on theoretical knowledge, clinical experience and intuition.
Rollison et al. (2021) Redeployment of Certified Registered Nurse Anesthetists During the Coronavirus Disease 2018 pandemic AANA Journal USA	To demonstrate the impact of Pandemic on nurse anesthetist Workforce	Short communication	n= 67 Certified Registered Nurse Anesthetists (CRNAs)	CRNAs were predominantly needed for ICU and respiratory therapist roles. CRNAs as respiratory therapists CRNAs as ICU nurses
Sanclemente-Dalmau et al. (2022) Defining competencies for nurse anaesthetists: A Delphi study Journal of Advanced Nursing Spain	Definition of competencies of nurse anaesthetist in the hospitals of Catalonia based on clinical practice through a consensus building process	Modified conventional delphi study Two rounds	n= 16 CRNAs	Final set of competencies consists of 139 items distributed among seven domains based on the framework of the IFNA
Schreiber and McDonald (2010) Keeping Vigil over the Patient: a grounded theory of nurse anaesthesia practice. Journal of Advanced Nursing Canada/USA	Exploration and development of a theory of nurse anesthesia practice in the USA. Exploration of the NA role, in the context of the nursing profession.	Grounded theory Constant comparative method	Interviews: practitioners, leaders and students (n= 18) Interviews (n= 41) of key informants Telephone interviews (n= 11)	To “keep vigil” CRNAs use 4 strategies which are connected with each other: Engaging with the patient Finessing the human-technology interface Massaging the message Foregrounding nursing
Sundqvist and Carlsson (2014) Holding the patient's life in my hands: Swedish registered nurse anaesthetists’ perspective of advocacy. Scand J Caring Sci Sweden	Description of advocacy in anesthesia care during the perioperative phase	Qualitative explorative design Inductive qualitative content analysis	n= 20 NAs, 2 Swedish hospitals Purposive maximum variation sample	Main theme**:** holding the patient's life in my hands, 3 subthemes Providing dignified care Providing safe care A moral commitment
Sundqvist et al. (2018) Promoting person-centred care in the perioperative setting through patient advocacy: an observational study. Journal of Clinical Nursing Sweden	Examination if the findings from an integrative review regarding perioperative patient advocacy could be empirically supported.	Qualitative descriptive observational study Content analysis	Registered nurse anesthetists n= 8, county hospital Sweden	Empirical support of the categories derived from an integrative review Identification of 11 new subcategories Main categories: Protecting Value preserving Supporting Informing

Abbreveations:

APN= advanced practice nurse

ASA= American Society of Anesthesiologists

CanMEDS= Canadian Medical Education Directives for Specialists

CRNA= certified registered nurse anesthetist

ICU= intensive care unit

IFNA= International Federation of Nurse Anesthetists

n.a.= not applicable

NA= nurse anesthetist

OR= operating room

PACU= post anesthesia care unit

PAN= perianesthesia nurses

RN= registered nurse

SD= standard deviation

US= United States

To synthesize the data from the results, discussion, and conclusion sections of the articles, qualitative content analysis (Kuckartz, 2018) was performed in MAXQDA2020©. Six out of the seven main categories were deduced from the framework of the International Federation of Nurse Anesthetists (2016), while one category emerged from the included publications. The main themes were supplemented by eleven inductive categories originating from the articles. The research team developed and revised the categories cooperatively. At first, all articles were read, and subsequently, approximately 30 % of the material was coded with the main categories. Subcategories were identified in parallel. After this process, we refined the category system and coded all included publications accordingly.

### 2.5 Registration

We registered the integrative review at PROSPERO on 15.08.2022 with the registration number CRD42022351430.

## 3 Results

We included 26 publications that either directly or indirectly addressed the skills and competencies of nurse anesthetists. Of these, eight and ten publications were of qualitative and quantitative natures, respectively, one was a mixed methods design, and seven were scientific reports.

The studies were conducted in Sweden (n= 8), Denmark (n= 1), Norway (n= 1), China (n= 1), Japan (n= 1), South Korea (n= 1), the USA (n= 7), Spain (n= 1), Iran (n= 1), Canada (n= 1), UK (Northern Ireland) (n= 1), Europe (n= 1), and Switzerland (n= 1). We extracted the following main themes from the included publications: professional competencies, communication competencies, coordination and management competencies, scholarship competencies, advocacy competencies, collaboration competencies, and clinical competencies. The main themes were supplemented by eleven inductive sub-themes derived from the included publications. The main themes and sub-themes are displayed in Table 2 and summarized below. Table 3 presents the technical skills performed by nurse anesthetists as well as their level of autonomy.

**Table 2:** Data synthesis main-themes and sub-themes.

**Main themes**	**Sub themes**	**Description**	**Sources**
**Professional competencies** **n = 11**		Being a role model Advance care by being innovative Create public, organizational and political awareness of the NA role Demonstrate expertise Stand up for the NA profession to expand scope of practice Membership in a professional association The COVID-19 pandemic as an opportunity for the profession Maintaining moral and ethical standards, treating patients equally and with respect, preserving integrity	(Everson et al., 2021; Federico, 2007; Halakou et al., 2017; Herion et al,. 2019; Matsusaki and Sakai, 2011; Neft et al., 2013; Rollison et al., 2021; Schreiber and MacDonald, 2010; Sanclemente-Dalmau et al., 2022; Sundqvist and Carlsson, 2014; Sundqvist et al., 2018)
**Communication competencies** **(n = 10)**	Patient communication (n= 7)	Advanced communication skills to: Establish patient relationships in the shortest possible time Gain the confidence and trust of patients through verbal and non-verbal communication obtain information, e.g. regarding medical history, risk factors Perioperative dialogue to help patients cope with fear	(Abelsson et al., 2021; Averlid and Høglund, 2020; Dahlberg et al., 2022b); (Herion, et al., 2019; Olin et al., 2022; Rönnberg et al., 2019; Sanclemente-Dalmau et al., 2022; Schreiber and MacDonald, 2010; Sundqvist and Carlsson, 2014; Sundqvist et al., 2018)
	Interprofessional communication (n= 5)	Advanced communication skills: As a prerequisite for good working relationships To deliver essential information in a short amount of time To resolve conflicts	
**Coordination/** **management competencies** **(n = 15)**	Anesthetic care process (n= 10)	Development of care plans based on scientific evidence, knowledge, protocols, and clinical skills Plan, implement and evaluate care based on anesthetic assessment Consider individual patient characteristics and risks factors Development of anesthesia plans under the supervision of anesthesiologists	(Callan et al., 2021; Chen et al., 2020; Olin et al., 2022; Dahlberg et al., 2022b; Everson et al., 2021; Federico, 2007; Fynes et al., 2014; Halakou et al., 2017; Herion et al., 2019; Ide et al., 2020; Rönnberg et al., 2019; Schreiber and MacDonald, 2010) (Sanclemente-Dalmau et al., 2022; Sundqvist and Carlsson, 2014; Sundqvist et al., 2018)
	Task management (n= 9)	The Operating Room (OR) as a complex and dynamic setting for which management competencies are required The ability to multitask is pivotal, as nurse anesthetists are frequently interrupted by various causes Organize materials, allocate resources, prepare, and check equipment Ensure high quality care	
**Scholarship competencies** **(n = 16)**	Research (n= 6)	Nurse anesthetists are skilled in research and research implementation Relevance of science in clinical practice varies between studies For clinically practicing nurse anesthetists, research seems to play a subordinate role	(Averlid and Høglund, 2020; Callan et al., 2021; Chen et al., 2020; Dahlberg et al., 2022a; Dahlberg et al., 2022b; Everson et al., 2021; Federico, 2007; Fynes et al., 2014; Halakou et al., 2017; Herion et al., 2019; Neft et al., 2013; Olin et al., 2022; Rollison et al., 2021; Rönnberg et al., 2019; Schreiber and McDonald, 2010; Sanclemente-Dalmau et al., 2022)
	Mentorship (n= 8)	Balancing a dual role as a nurse anesthetist and mentor in the OR can be challenging Mentoring, teaching, supervising, educating, and sharing knowledge with students and colleagues intra- and interprofessionally	
	Knowledge (n= 14)	Nurse anesthetists have extensive clinical knowledge in diverse fields of practice and are able to provide advanced care in the OR and beyond Clinical decision-making based on advanced knowledge, experiences, and intuition Nurse anesthetists’ knowledge of advanced life support, airway and respiratory management, emergencies etc. is a resource for the healthcare team and allowed them to take on different roles during the COVID-19 pandemic Nurse anesthetists are committed to lifelong learning The following educational needs could be identified: pedagogics mentoring academic knowledge business management	
**Advocacy competencies** **(n = 10)**	Protect patient autonomy (n= 8)	Preserving patient autonomy is an integral part of high quality anesthetic care which comprises of: Empowerment through patient education Giving the patient the opportunity to decide and participate in care Ensuring that wishes of patients are met even in an unconscious state Advocacy can be burdensome and cause moral distress	(Abelsson et al., 2021; Dahlberg et al., 2022b; Fynes et al., 2014; Herion et al., 2019; Ide et al., 2020; Rönnberg et al., 2019; Schreiber and MacDonald, 2010; Sundqvist and Carlsson, 2014; Sundqvist et al., 2018; Sanclemente-Dalmau et al., 2022)
	Acting on behalf of the patient (n= 7)	Nurse anesthetists feel obliged to: Protect the patients from harm while unconscious Act as surrogate by taking over vital body functions Ensure that the best outcomes are achieved Stand up for the patients	
**Collaboration competencies** **(n = 16)**	Leadership (n= 4)	Nurse anesthetists assumed leadership positions in the course of the pandemic Knowledge and leadership skills are important, as the OR is a dynamic environment which necessitates a flexibility in leadership styles Taking over leadership roles to overcome practice barriers	(Callan et al., 2021; Dahlberg et al., 2022b; Everson et al., 2021; Federico, 2007; Fynes et al., 2014; Halakou et al., 2017; Herion et al., 2019; Neft et al., 2013; Olin et al., 2022; Rayborn et al., 2017; Rollison et al., 2021; Rönnberg et al., 2019; Sanclemente-Dalmau et al., 2022; Schreiber and MacDonald, 2010; Sundqvist and Carlsson, 2014; Sundqvist et al., 2018)
	Teamwork (n= 10)	Close intra- and interprofessional collaboration The healthcare team works jointly towards a common goal	
	Conflicts in the healthcare team (n= 5)	Interprofessional competition may lead to conflicts between nurse anesthetists and anesthesiologists Hierarchical structures can impair collaboration Conflicts as a possible limiter of scope of practice Dealing with conflicting opinions related to patient care	
**Clinical competencies (n=24)**	Technical skills (n= 15)	Technical skills performed differ at national and international level Scope of practice and technical skills have expanded as a result of the pandemic	(Callan et al., 2021; Chen et al., 2020) (Dahlberg et al., 2021; Dahlberg et al., 2022a; Dahlberg et al., 2022b; Egger Halbeis and Schubert, 2008; Everson et al., 2021; Federico, 2007; Fynes et al., 2014; Halakou et al., 2017; Herion et al., 2019; Ide et al., 2020; Lauridsen et al., 2015; Matsusaki and Sakai, 2011; Meeusen et al., 2010; Neft et al., 2013; Olin et al., 2022; Rayborn et al., 2017; Rollison et al., 2021; Rönnberg et al., 2019; Schreiber and MacDonald, 2010; Sundqvist and Carlsson, 2014; Sundqvist et al., 2018; Sanclemente-Dalmau et al., 2022)
	Level of autonomy (n= 17)	One can distinguish between independent practice, direct supervision and indirect supervision Scope of practice and level of autonomy is influenced by politics, legal regulations, organizational policy, history of the nursing profession, culture, anesthetists, education, degree, the COVID-19 pandemic, workforce shortages, American Society of Anesthesiologists status of patients CRNAs may practice independently according to hospital guidelines, protocols, or anesthesiologists order Restrictions in scope of practice are common Expanded scope of practice and higher autonomy can lead to higher job satisfaction, higher levels of experience and skills Different educational levels and job titles could be identified	
	Patient safety and risk management (n= 9)	Advanced skills, knowledge, and experience in the management of emergencies Nurse Anesthetists ensure patient safety in all anesthesia phases through adhering to standards, protocols, and checklists To ensure patient safety they need to be proactive, one step ahead, anticipate risks, use risk assessment tools and have several contingency plans Early signs of deterioration need to be recognized and managed adequately	

Abbreveations:

ASA= American Society of Anesthesiologists

CRNA= certified registered nurse anesthetist

OR= operating room

NA= nurse anesthetist

**Table 3:** Clinical skills.

**Scope of practice**	**Skill**	**Level of autonomy**		
		**Not specified**	**Collaborative/** **under supervision**	A**utonomous**
Before Procedure	Pre anesthesia visits (assessment) n= 7	(Fynes et al., 2014; Ide et al., 2020; Matsusaki and Sakai, 2011; Rayborn, et al., 2017)	(Sanclemente-Dalmau et al., 2022; Chen et al., 2020; Meeusen et al., 2010)	(Sanclemente-Dalmau et al., 2022)
	Assess anesthetic risks n= 2	(Sanclemente-Dalmau et al., 2022)		(Rönnberg et al., 2019)
	Obtain informed consent n= 1	(Rayborn et al., 2017)		
	Develop anesthesia plans n= 4	(Matsusaki and Sakai, 2011; Rayborn et al., 2017)	(Chen et al., 2020)	(Rönnberg et al., 2019)
	Airway assessment n= 2	(Rollison, et al., 2021; Rönnberg et al., 2019)		
	Check equipment n= 1	(Rayborn et al., 2017)		
Intra-operative	Induce general anesthesia ASA I and II patients n= 1			(Meeusen et al., 2010)
	Non-invasive airway management n= 3	(Everson et al., 2021; Matsusaki and Sakai, 2011)		(Sanclemente-Dalmau et al., 2022)
	Invasive airway management (laryngeal mask orotracheal intubation) n= 3	(Callan et al., 2021; Everson et al., 2021)	(Sanclemente-Dalmau et al., 2022)	
	Airway handling/ management not specified n= 3	(Rayborn et al., 2017; Callan et al., 2021)	(Lauridsen, et al., 2015)	
	Insert/remove oropharyngeal airway n= 1			(Dahlberg et al., 2022a)
	Insert/remove nasopharyngeal airway n= 1			(Dahlberg et al., 2022a)
	Intubation n= 5	(Callan et al., 2021; Everson et al., 2021; Rollison et al., 2021; Fynes et al., 2014; Rayborn et al., 2017)		(Everson et al., 2021)
	Intubation with video laryngoscopy n= 1		(Ide et al., 2020)	
	Remove laryngeal mask n= 1			(Dahlberg et al., 2022a)
	Patient positioning n= 1	(Everson et al., 2021)		
	Bag valve mask ventilation n= 1			(Dahlberg et al., 2022a)
	Respiratory treatments n= 1	(Callan et al., 2021)		
	Ventilator management/care (adjustment of respiratory modes, setting up parameters) n= 4	(Callan et al., 2021; Everson et al., 2021)		(Chen et al., 2020; Dahlberg et al., 2022a)
	Insert intravenous lines n= 1		(Ide et al., 2020)	
	Respiratory support n= 1	(Callan et al., 2021)		
	Chest tube placement n= 1	(Callan et al., 2021)		
	Anesthesia induction n= 1	(Callan et al., 2021)		
	Neuraxial blocks n= 1	(Callan et al., 2021)		
	Peripheral nerve blocks n= 2	(Callan et al., 2021; Rayborn et al., 2017)		
	Provide regional anesthesia n= 2	(Everson et al., 2021) (Rayborn et al., 2017)		
	Administer anesthesia n= 2		(Egger Halbeis and Schubert, 2008)	(Everson et al., 2021)
	Monitoring n= 6	(Callan et al., 2021; Chen et al., 2020; Matsusaki and Sakai, 2011; Sanclemente-Dalmau et al., 2022)		(Dahlberg et al., 2022a; Dahlberg et al., 2021)
	Monitoring and interpreting electrocardiograms n= 1			(Dahlberg et al., 2022a)
	Select and administer anesthetic and adjuvant drugs n= 2	(Matsusaki and Sakai, 2011; Rayborn et al., 2017)		
	Administer opioids n= 1			(Dahlberg et al., 2022a)
	Administer anti-emetics n= 1			(Dahlberg et al., 2022a)
	Central line placement n= 3	(Callan et al., 2021; Everson et al., 2021)	(Dahlberg et al., 2021)	
	Arterial line placement n= 4	(Callan et al., 2021; Everson et al., 2021)	(Dahlberg et al., 2021)	(Dahlberg et al., 2022a)
	Injections into an epidural catheter n= 1		Depending on country (Dahlberg et al., 2021)	Depending on country (Dahlberg et al., 2021)
	Connecting and adjusting pacemakers n= 1	(Dahlberg et al., 2021)		
	Extubation n= 2	(Rönnberg et al., 2019)	(Chen et al., 2020)	
	Decide when to extubate n= 1	(Rayborn et al., 2017)		
	Emergence from anesthesia n= 4	(Callan et al., 2021; Matsusaki and Sakai, 2011; Rayborn et al., 2017; Rönnberg et al., 2019)		
Post-operative	Postanesthesia care/management n= 4	(Rayborn et al., 2017) (Halakou et la., 2017)	(Chen et al., 2020; Dahlberg et al., 2022a)	
	Administer appropriate drugs n= 1		(Sanclemente-Dalmau et al., 2022)	
	Discharge patients n= 2	(Matsusaki and Sakai, 2011)	Depending on country (Dahlberg et al., 2021)	Depending on country(Dahlberg et al., 2021)
	Patient transfer n= 2	(Chen et al., 2020) (Sanclemente-Dalmau et al., 2022)		
Other services	Advanced Life Support n= 6	(Chen et al., 2020; Dahlberg et al., 2022b; Herion et al., 2019; Matsusaki and Sakai, 2011; Fynes et al., 2014) (Sanclemente-Dalmau et al., 2022)		
	Basic Life Support n= 1	(Matsusaki and Sakai, 2011)		
	Bronchoscopy n= 1	(Callan et al., 2021)		
	Chest tube placement n= 1	(Callan et al., 2021)		
	Prescribing medication n= 2	(Neft et al., 2013)(Sanclemente-Dalmau et al., 2022)		
	Respiratory support n= 1	(Callan et al., 2021)		
	Handle and check equipment (e.g. respirators) n= 1	(Rollison et al., 2021)		
	Caring for COVID-19 patients n= 1		(Rollison et al., 2021)	
	Cardiac arrest/rapid response team n= 2	(Rollison et al., 2021)	(Lauridsen et al., 2015)	
	Sedation (for procedures) n= 2	(Ide et al., 2021)	(Meeusen et al., 2010)	
	Documentation n= 3	(Rollison et al., 2021) (Rayborn et al., 2017; Herion et al., 2019)		
	Pain management n= 3	(Ide et al., 2020) (Rayborn et al., 2017; Herion et al., 2019)		

Not specified= not clearly stated in the article

Collaboration/supervision= skill is performed collaboratively or under direct or indirect supervision of a physician

Autonomous= skill is performed independently

Abbreviation: ASA= American Society of Anesthesiologists

### 3.1 Professional competencies (n= 11)

As professionals, nurse anesthetists advance care through innovation and advocate for the profession, both publicly and in the workplace, to expand and defend their scope of practice (Everson et al., 2021; Neft, et al., 2013; Rollison et al., 2021). The importance of competencies in the maintenance of moral and ethical standards as well as the protection and defense of the patients’ integrity and rights was mentioned by Halakou et al. (2017), Sundqvist and Carlson (2014) and Sundqvist et al. (2018). Serving as a role model is defined as part of nurse anesthetists’ competencies by Halakou et al. (2017) as well as Herion et al. (2019).

### 3.2 Communication competency (n= 10)

Ten articles addressed communication competencies, from which we extracted the two inductive themes “interprofessional communication” (n= 5) and “patient communication” (n= 7).

#### 3.2.1 Interprofessional communication (n= 5)

Within the interprofessional team, advanced communication skills are a prerequisite for facilitating a good working climate and are essential for resolving conflicts, delivering vital information, and achieving favorable patient outcomes (Dahlberg et al., 2022b; Olin et al., 2022; Schreiber and MacDonald, 2010; Sundqvist et al., 2018).

#### 3.2.2 Patient communication (n= 7)

Sanclemente-Dalmau et al. (2022) describe the communicator role as part of nurse anesthetists’ competencies, including effective communication skills with patients as well as families and providing emotional support to both parties. Nurse anesthetists, therefore, have to establish a relationship with the patient in the shortest possible time and gain trust through verbal and non-verbal communication (Abelsson et al., 2021; Dahlberg et al., 2022b; Rönnberg et al., 2019; see Table 2 for detailed references). In the form of the perioperative dialogue, nurse anesthetists inform patients about the anesthesia process in order to help them cope and take control of the situation (Abelsson et al., 2021; Sanclemente-Dalmau et al., 2022). As experts, nurse anesthetists perform pre-anesthesia visits, prepare patients for and accompany them throughout the anesthesia process, and provide information to ensure patient understanding (Sanclemente-Dalmau et al., 2022). According to Abelsson et al. (2021), nurse anesthetists support patients through the perioperative dialogue to help them cope with fear, by focusing on their needs and experiences and distracting them through conversation and physical touch.

### 3.3 Coordination and management competencies (n= 15)

In 15 publications, coordination and management competencies were addressed from which the two subcategories “anesthetic care process” (n= 10) and “task management” (n= 9) were derived.

#### 3.3.1 Anesthetic care process (n= 10)

As part of the anesthetic care process, nurse anesthetists develop care plans based on scientific evidence (Sanclemente-Dalmau et al., 2022), knowledge, protocols, and skills, taking into account the individual patient's characteristics and risk factors (Federico, 2007; Dahlberg et al., 2022b). They develop anesthesia plans under the supervision of anesthesiologists (Chen et al., 2020) and, in some countries, perform pre-anesthetic assessments (Fynes et al., 2014).

#### 3.3.2 Task management (n= 9)

Task management encompasses the selection and preparation of equipment and medication, allocation and efficient use of resources, and prioritization of tasks (Callan et al., 2021; Dahlberg et al., 2022b; Everson et al., 2021; see Table 2 for additional references). As the operating room is a complex and dynamic setting, the ability to multitask, organize, and deal with frequent interruptions is necessary to ensure and maintain high-quality patient care (Olin et al., 2022).

### 3.4 Scholarship competencies (n= 16)

The sub-themes research (n= 6), mentorship (n= 8), and knowledge (n= 14) were extracted from the literature based on 16 articles that referred to scholarship competencies.

#### 3.4.1 Research (n= 6)

Participation in research and incorporation of research findings into daily practice was described as part of nurse anesthetists’ competencies (Halakou et al. 2017; Herion et al., 2019; Federico, 2007; see Table 2 for additional references). Nurse anesthetists addressed the importance of academic competence with regard to providing theoretical guidance to students (Averlid and Høglund, 2020).

#### 3.4.2 Mentorship (n= 8)

Mentoring in anesthesia care requires nurses to fulfill a dual role, as they need to find a balance between patient safety and the learning needs of students. An integral part of mentoring is helping students to gain confidence and cope with stressful situations (Averlid and Høglund, 2020). Supervising novice nurses, physicians, and other healthcare professionals is also part of the nurse anesthetist-mentoring role (Dahlberg et al., 2022b; Everson et al., 2021; Federico, 2007; Fynes et al., 2014). During the COVID-19 pandemic, nurse anesthetists provided guidance on an interprofessional level and were consulted concerning sedation or airway management (Rollison et al., 2021). Therefore, advanced skills in mentoring are essential to fulfil the demanding role of a mentor in anesthesia care (Averlid and Høglund, 2020).

#### 3.4.3 Knowledge (n= 14)

In the 14 publications wherein knowledge was addressed, we included statements referring to essential knowledge for practicing as a nurse anesthetist as well as knowledge gaps.

The COVID-19 pandemic allowed nurse anesthetists not only to expand their scope of practice but also to demonstrate and share their advanced knowledge and skills in diverse fields of practice. Healthcare professionals benefited from nurse anesthetists’ knowledge in advanced airway management, airway and respiratory emergencies, resuscitation, and advanced life support (Chen et al., 2020; Rollison et al., 2021). Halakou et al. (2017) and Herion et al. (2019) describe that nurse anesthetists are required to be committed to life-long learning and professional development. When it comes to extubation, for example, it is the combination of extensive knowledge and experience that guides nurse anesthetists in decision-making (Rönnberg et al., 2019).

Regarding mentoring, in a study by Averlid and Høglund (2020), participating nurse anesthetists identified pedagogic training and academic competence as important educational aims.

### 3.5 Advocacy competencies (n= 10)

Ten articles contained aspects of advocacy, which is defined by Bu and Jezewski (2007) as the protection of autonomy, taking actions on behalf of the patient, and taking a stand for social justice in health care. The two inductive sub-themes, “acting on behalf of the patient” and “protecting patient autonomy”, were named according to the definition provided by Bu and Jezewski (2007).

#### 3.5.1 Acting on behalf of the patient (n= 7)

Unconscious patients are reported to be in a vulnerable state, and by taking over vital body functions, nurse anesthetists hold, as described by Sundqvist and Carlsson (2014), the lives of patients in their hands. Nurse anesthetists feel obliged to represent and protect patients’ interests (Abelsson et al., 2021). To do so, they take on a surrogate role (Schreiber and MacDonald, 2010). If they feel that these interests are contradicted by other healthcare professionals or procedures, they feel committed to intervene (Rönnberg et al., 2019; Schreiber and MacDonald, 2010; Sundqvist and Carlsson, 2014 see Table 2 for additional references).

#### 3.5.2 Protecting patient autonomy (n= 8)

Nurse anesthetists safeguard patients’ autonomy through diverse strategies. To empower patients and mitigate the fear of loss of control, they provide comprehensible information that fosters the patient's capability for informed decision-making and participation (Abelsson et al., 2021; Schreiber and MacDonald, 2010). As described by Sundqvist et al. (2018), the autonomy of individuals can be promoted through small actions, such as assuming a position at the same level as the patients, considering patients’ preferences regarding the position on the operating room table, and providing information about what to expect next.

### 3.6 Collaboration competencies (n= 16)

In 16 publications, collaboration competencies were addressed and three inductive sub-themes were derived from the literature.

#### 3.6.1 Teamwork (n= 10)

The nurse anesthetist's role as a collaborator is described as one of promoting the effective cooperation and professional relationships between different healthcare providers in the operating room and beyond (Dahlberg et al., 2022b; Federico, 2007; Herion et al., 2019; Sanclemente-Dalmau et al., 2022). Teamwork encompasses collaboration with different healthcare professionals to foster innovation, overcome barriers or problems, and advance patient care, and it involves collaborating in interdisciplinary research as well as educational activities (Herion et al., 2019; Sanclemente-Dalmau et al., 2022). According to Olin et al. (2022), interactions with other healthcare providers in the operating room most frequently occurred during preparation for the induction of anesthesia, the induction phase, and during extubation. When it comes to collaboration, nurse anesthetists take on coordinating roles within the healthcare team.

#### 3.6.2 Conflict management (n= 5)

Because of overlapping competencies, conflicts, ambiguity, and competition may arise between nurse anesthetists and anesthesiologists, as described by Rayborn et al. (2017). Furthermore, balancing time pressure and patient safety can lead to discussions and the feeling of being questioned by other team members. Thus, nurse anesthetists need to have the assertiveness to defend their decisions (Rönnberg et al., 2019), an awareness of overlapping functions and sources of tension, as well as knowledge of resolution strategies (Herion et al., 2019). Schreiber and MacDonald (2010) describe the emergence of a power play or manipulation between nurse anesthetists and anesthesiologists if conflicts cannot be resolved immediately. Nurse anesthetists must deal with conflicting opinions, feelings of isolation, and a lack of respect from other healthcare professionals (Sundqvist and Carlsson, 2014).

#### 3.6.3 Leadership (n= 4)

The participants in the study of Halakou et al. (2017) agreed upon nurse anesthetists’ independent supervisory management of the anesthesia unit, the operating room, as well as pain management departments, and the importance of decision-making abilities. Herion et al. (2019) connect leadership to the dissemination of research, participation in national organizations, and awareness of policy issues. In two publications, motivational skills were mentioned (Halakou et al., 2017; Herion et al., 2019). During the COVID-19 pandemic, nurse anesthetists assumed leadership positions (Everson et al., 2021).

### 3.7 Clinical competencies (n= 24)

Twenty-four publications addressed clinical competencies, from which we extracted three sub-themes: technical skills (n= 15), level of autonomy (n= 17), and patient safety and risk management (n= 9). A detailed list of the identified technical skills is displayed in Table 3. The skills were subsumed under the scope of practice of nurse anesthetists, as defined by the International Federation of Nurse Anesthetists (2016) as well as the International Council of Nurses (2021), and categorized according to the level of nurse anesthetists’ autonomy. If the degree of autonomy was not clearly stated in the article, we assigned the label “not clearly specified”.

#### 3.7.1 Technical skills (n= 15)

Nurse anesthetists perform a wide variety of clinical skills in all anesthesia phases as well as in other services (Fynes et al., 2014; Herion et al., 2019; Matsusaki and Sakai, 2011; Sanclemente-Dalmau et al., 2022, see table 3 for additional references). In several publications, nurse anesthetists provide sedation for procedures (Ide et al., 2020; Meeusen et al., 2010) or perform pain management activities (Ide et al., 2020; Rayborn et al., 2017; Herion et al., 2019). As stated by Rönnberg et al. (2019), nurse anesthetists in Sweden administer anesthesia to patients of American Society of Anesthesiologists status I and II, but under indirect supervision of an anesthesiologist. The authors further describe extubation performed by nurse anesthetists as a complex task involving clinical decision-making, intuition, and experience. One study participant defined extubation as an art form (Rönnberg et al., 2019). In Japan, perianesthesia nurses are assigned to American Society of Anesthesiologists status I and II; however, during induction and emergence from anesthesia, the presence of an anesthesiologist is mandatory. In some US states, the COVID-19 pandemic led to an expanded scope of practice of nurse anesthetists predominantly outside the operating room. Nurse anesthetists performed tracheal intubation as well as arterial and central line placement (Callan et al., 2021).

#### 3.7.2 Level of nurse anesthetist autonomy (n= 17)

According to Meeusen et al. (2010), different types of supervision exist. Autonomous practice, direct supervision, and indirect supervision are distinct categories that can be differentiated. Direct supervision, as defined by Meeusen et al. (2010), refers to the constant presence of an anesthesiologist, while indirect supervision allows the anesthesiologist's absence from the room but immediate availability for assistance. Regarding pre-anesthesia visits, Chen et al. (2020) and Meeusen et al. (2010) characterize them as collaborative tasks. However, in four publications, the level of autonomy remains unspecified. Invasive airway management is considered a clinical skill of nurse anesthetists by three authors, and Sanclemente-Dalmau et al. (2022) state that it is practiced collaboratively. Five authors mention nurse anesthetists performing intubations, but in four publications, the level of autonomy is not specified. Nevertheless, Everson et al. (2021) indicate that nurse anesthetists took on intubation responsibilities due to the pandemic. These examples demonstrate variations in the level of autonomy among nurse anesthetists across the included publications and that the level of autonomy is seldom clearly specified (Callan et al., 2021; Chen et al., 2020; Dahlberg et al., 2021; Dahlberg et al., 2022a; see Table 3 for additional references).

#### 3.7.3 Patient safety and risk management (n= 9)

Nurse anesthetists practice in accordance with standards and protocols and adhere to checklists and assessment instruments to ensure patient safety (Herion et al., 2019; Sanclemente-Dalmau et al., 2022). Incorporating contingency plans and keeping emergency equipment readily available helps nurse anesthetists to stay ahead of potential risks (Rönnberg et al., 2019; Sundquvist and Carlsson, 2014).

## 4 Discussion

The International Federation of Nurse Anesthetists (2016) defines nurse anesthetists as being experts, communicators, collaborators, managers, health advocates, scholars, and professionals, all of which coincide with the competencies identified through our review. Additionally, we extracted several clinical competencies of nurse anesthetists that reveal the advanced set of skills performed by nurse anesthetists in some countries and their broad scope of practice. However, heterogeneity in several areas could be identified in our review.

Similar to Austria, the development of the nurse anesthetist role in some countries, such as Iran and Spain, is still in an early stage. In these countries, the role itself is not yet legally regulated and competencies are not clearly differentiated (Halakou et al., 2017; Sanclemente-Dalmau et al., 2022). In Catalonia, Spain, the shift of general nursing education to universities as a bachelor's program resulted in nurse anesthetists being trained at the master's level. This led to an increase in competencies, though at an unregulated level (Sanclemente-Dalmau et al., 2022).

When considering the Austrian legal requirements for the education of anesthetic nurses, the discrepancies with international competency domains reveal a narrower competency framework for Austrian nurses working in the field of anesthesia and the need for an expansion of their education and scope of practice. Anesthesia care in Austria involves the observation, care, as well as the monitoring of patients before, during, and after anesthesia and assisting anesthesiologists in anesthesia procedures. Anesthetic nurses are authorized to draw arterial blood samples from existing catheters but are not permitted to insert them. They also participate in resuscitation and shock therapy, and monitor critically ill patients using invasive and non-invasive methods (Health and Nursing Act 1997, §20). As identified through our review, the provision of anesthesia to American Society of Anesthesiologist status I and II patients (Rönnberg et al., 2019; Ide et al., 2020) or tasks such as the insertion of central or arterial lines and tracheal intubation (Callen et al., 2021) fall outside the scope of practice of Austrian anesthetic nurses, and they do not meet the educational criteria for advanced practice nurses.

Nurse anesthetists, as defined by the International Council of Nurses (2021), are advanced practice nurses with a master's degree and, in contrast to specialist nurses, have an extended scope of practice. Similar to Spain, Austria is now transitioning towards a bachelor's degree in general nursing education, making it reasonable for Austria to implement a post-graduate master's level qualification for anesthetic nurses. Both in Norway and South Korea, two educational levels for nurse anesthetists are offered (Averlid and Høglund, 2020; Rayborn et al., 2017). In Norway, nurses can either complete a university program of 120 European Credit Transfer System Points to receive a master's degree in nurse anesthesia or complete a post-bachelor program of 90 European Credit Transfer System Points, which also qualifies them as nurse anesthetists (International Federation of Nurse Anesthetists, 2022). Averlid and Høglund (2020) discovered that the increase in students pursuing a master's degree exposed knowledge deficiencies among mentors in direct care, particularly regarding academic and pedagogical knowledge. In South Korea, two non-physician anesthesia roles coexist – anesthesia registered nurses and certified registered nurse anesthetists. According to Rayborn et al. (2017), there are statistically significant differences in both job satisfaction and components of practice between these two roles, with the scope of practice of certified registered nurse anesthetists pointing towards a more advanced practice. Such differences in the scope of practice as well as in shared responsibilities may lead to interprofessional competition, as described by Rayborn et al. (2017). Hence, in the Austrian context, it cannot be recommended to have a dual distribution of roles in anesthesia nursing.

When considering the role of research in anesthesia nursing, it becomes apparent that while research is listed among the competencies of nurse anesthetists by the International Federation of Nurse Anesthetists (2016), its relevance may not hold the same level of prominence across all included publications, given that this aspect was only addressed in four of the included publications. Nevertheless, the ability to conduct nursing research is pivotal for advancing the profession, substantiating the safety of nurse led anesthesia and addressing key questions in anesthesia care. In their study, Dulisse and Cromwell (2010) demonstrated nurse anesthetists’ ability to deliver high-quality and safe anesthesia care as they found no increase in patient mortality in US states where nurse anesthetists work independently. This aspect highlights the relevance of scientific competencies and the ability to conduct research for nurse anesthetists, as further high-quality studies focusing on patient outcomes are required to expand their scope of practice (Lewis et al., 2014; Vitale and Lyons, 2021).

The International Federation of Nurse Anesthetists (2016) describes nurse anesthetists as being health advocates, responsible for the preservation of patient rights, independence, decisions, and privacy. The results of our review support and expand the role of nurse anesthetists as advocates, emphasizing the high level of responsibility that they assume for their patients. We extracted two inductive sub-themes “acting on behalf of the patients” and “protecting autonomy” from the literature. These include protecting the patients’ interests and defending them, if they feel that these interests are violated (Abelsson et al., 2021). In doing so, they assume a proxy role. This can be interpreted in the light of substitutive and moral/ethical advocacy as described in the study of Kubsch et al. (2004). While the patients are in an unconscious state, nurse anesthetists try to balance out previously formulated decisions as well as medical and safety requirements to protect the interests of patients. As elaborated by Sundqvist and Carlsson (2014), advocacy in anesthesia nursing comprises diverse aspects, like standing up for the patient, providing care in accordance with guidelines, and giving understandable information. These findings demonstrate the relevance of non-technical skills for nurse anesthetists, which also apply to cooperating with the interprofessional team.

Regarding technical skills, it becomes apparent that there is a substantial variation in both the level of autonomy and individual skills across the included publications. As indicated by our review, the degree of autonomy concerning the performance of technical skills often lacks clarity in the publications included. However, precise information regarding the level of supervision and autonomy is relevant for policymakers and educators to develop curricula as well as legal requirements. This indicates that further research regarding the tasks performed by nurse anesthetists and the level of autonomy is required. The International Council of Nurses (2021), as well as the International Federation of Nurse Anesthetists (2016), have developed standards and graduate competencies aimed at improving the standards of practice and education, as well as expanding nurse anesthetists’ scope of practice. The Austrian nursing law is relatively strict compared to the US, where advanced nurse practitioners hold prescriptive authority (Dillon and Gary, 2017) or nurse anesthetists in opt-out states may practice without the supervision of a physician (Egger Halbeis and Schubert, 2008; Munday, 2023). Austrian nurses are authorized to prescribe wound dressings or incontinence materials but do not have prescriptive authority for medications (Health and Nursing Act, 1997 §15a). Hoyem et al. (2019) conclude that the main drivers limiting the scope of practice of nurse anesthetists in the USA are politics and professional interests. This also applies to Austria, where efforts to change this situation are impeded by the Austrian Medical Chamber (Standard 2022) and the Austrian Society of Anesthesiology, Resuscitation and Intensive Care (2018). This highlights the importance of nurse anesthetists advocating for their profession as a competency to expand their areas of practice as described by several authors (Everson et al., 2021; Neft et al., 2013; Rollison et al., 2021). During the COVID-19 pandemic, nurse anesthetists could broaden their range of professional responsibilities due to their advanced skills and knowledge in respiratory care, advanced life support, and airway management (Chen et al., 2020). This expansion occurred mostly outside the operating room and included activities such as tracheal intubation and arterial and central line placement, thus supporting the expansion of nurse anesthetists’ scope of practice and highlighting their capabilities and expertise (Callan et al., 2021).

Internationally, the anesthesia workforce and the provision of anesthesia services are challenged by increased surgical demand, demographic changes, aging anesthesiologists, production pressure, part-time work, and staffing shortfalls (Egger Halbeis and Schubert, 2008; Haller et al., 2021). To cope with these system strains, across many countries, anesthesia is performed by physician as well as non-physician providers like nurse anesthetists, with the latter either substituting for or complementing anesthesiologists (Egger Halbeis and Schubert, 2008). Nurse anesthetists contribute to healthcare systems by delivering anesthesia services across 107 countries (International Council of Nurses, 2021). Austria is currently facing a scarcity of anesthesiologists (Markstaller, 2019), necessitating an exploration of alternative models for anesthesia provision. In this context, the implementation of nurse anesthetists should be proposed, as the international literature reports no significant differences in outcomes between physician and nurse-led anesthesia (Hoyem et al., 2019; Yin et al., 2021) Therefore, this discussion should be encouraged to balance shortages and ensure the safe delivery of anesthesia.

## 5 Limitations

Due to the authors’ limited foreign language skills, only studies published in English and German language were included. We decided to restrict the search period to the last 15 years, and despite seeking input from experts and a librarian, it is possible that some search terms might have been overlooked. Consequently, there is the possibility that other relevant publications might not have been identified. As the included literature originated from various countries, covered different stages of role development, and utilized different methodologies, the generalizability of the results is limited. However, as the aim of this integrative review was to explore the current international scope of practice, roles, and skillsets, heterogeneity was expected. To ensure rigor we followed the steps of conducting an integrative review according to Whittemore and Knafl (2005), critically appraised the included publications, and synthesized the data with qualitative content analysis according to Kuckartz (2018).

## 6 Conclusion

Nurse anesthetists exhibit a comprehensive set of competencies encompassing technical as well as non-technical skills, which enable them to provide high-quality and advanced anesthesia care for the benefit of their patients. Our findings emphasize the importance of advanced communication, management, scholarship, advocacy, collaboration, and clinical competencies of nurse anesthetists, as well as the need for these professionals to work towards an expansion of their scope of practice. As identified through our review, there are variations in the scope of practice, roles, and skills of nurse anesthetists across different countries. Consequently, in some countries, nurse anesthetists practice within a broad scope, while in others, often due to policy considerations, their responsibilities and skills are limited. The findings of our review can serve as a resource for Austria as well as other countries seeking to develop an advanced nursing role in anesthesia care in order to cope with diverse system strains. Further research aimed at identifying the technical skills executed by nurse anesthetists and clarifying the level of supervision during their performance could prove valuable in role development.

## Declaration of generative AI and AI-assisted technologies in the writing process

During the preparation of this work, the authors used DeepL in order to improve language and readability. After using this tool/service, the authors reviewed and edited the content as needed and take full responsibility for the content of the publication.

## Funding

This research did not receive any specific grant from funding agencies in the public, commercial, or not-for-profit sectors.

## CRediT authorship contribution statement

**Kathrin Julia Pann:** Writing – review & editing, Writing – original draft, Visualization, Validation, Methodology, Investigation, Formal analysis, Conceptualization, Data curation. **Verena-Katrin Buchner:** Writing – review & editing, Validation, Methodology, Conceptualization. **Roland Eßl-Maurer:** Writing – review & editing, Validation, Methodology, Conceptualization. **Tobias Bacher:** Writing – review & editing, Visualization, Validation, Methodology, Conceptualization. **Manela Glarcher:** Writing – review & editing, Visualization, Validation, Supervision, Project administration, Methodology, Conceptualization. **Andre Ewers:** Writing – review & editing, Validation, Supervision, Resources, Project administration, Methodology, Conceptualization.

## Declaration of competing interest

The authors declare that they have no known competing financial interests or personal relationships that could have appeared to influence the work reported in this paper.

## Appendix Supplementary materials

Image, application 1

Image, application 2
