# Supplementary material for: International competencies of nurses with advanced practice in anesthesia nursing: An integrative review
Source: Int J Nurs Stud Adv. 2025 Mar 17;8:100319. doi: 10.1016/j.ijnsa.2025.100319 (PMC11984995; doi:10.1016/j.ijnsa.2025.100319)
Supplement: Supplementary file 1 [file mmc1.docx]

Supplement 1: Search String PubMed

(("nurse practitioners"[MeSH Terms] OR "clinical nurse consultant*"[Title/Abstract] OR "nurse clinicians"[MeSH Terms] OR "clinical nurse specialist*"[Title/Abstract] OR "nurse practitioner*"[Title/Abstract] OR "nurse clinician*"[Title/Abstract] OR "nurse anesthetists"[MeSH Terms] OR "advanced practice nurse*"[Title/Abstract] OR "anesthesia nurs*"[Title/Abstract] OR "anaesthesia nurs*"[Title/Abstract] OR "certified registered nurse anesthetist*"[Title/Abstract] OR "certified registered nurse anaesthetist*"[Title/Abstract] OR "CRNA"[Title/Abstract] OR "nurse anesthetist*"[Title/Abstract] OR "nurse anaesthetist*"[Title/Abstract] OR "advanced practice registered nurs*"[Title/Abstract] OR "nurse anesthesiologist*"[Title/Abstract] OR (("nurse's"[All Fields] OR "nurses"[MeSH Terms] OR "nurses"[All Fields] OR "nurse"[All Fields] OR "nurses s"[All Fields]) AND "anaesthesiologist*"[Title/Abstract]) OR "anesthetic nurs*"[Title/Abstract] OR "anaesthetic nurs*"[Title/Abstract]) AND ("advanced nursing practice"[Title/Abstract] OR "advanced care"[Title/Abstract] OR "advanced nursing"[Title/Abstract] OR "Advanced Practice Nursing"[Title/Abstract] OR "scope"[Title/Abstract] OR "Scope of Practice"[Title/Abstract] OR "Scope of Practice"[MeSH Terms] OR "Nurse's Role"[MeSH Terms] OR "Professional Competence"[MeSH Terms] OR "Advanced Practice Nursing"[MeSH Terms] OR "skill*"[Title/Abstract] OR "postanesthesia nursing"[MeSH Terms] OR "perioperative care"[MeSH Terms] OR "perioperative nursing"[MeSH Terms] OR "anesthesia/nursing"[MeSH Terms] OR "practice patterns, nurses'"[MeSH Terms] OR "competence*"[Title/Abstract] OR "practice patter*"[Title/Abstract] OR "nurse s role*"[Title/Abstract] OR ("nurs*"[All Fields] AND "role*"[Title/Abstract]) OR "role*"[Title/Abstract] OR "professional competenc*"[Title/Abstract] OR "knowledge"[Title/Abstract] OR "clinical competence"[MeSH Terms] OR "emergency care"[Title/Abstract] OR "perioperative care"[Title/Abstract] OR "post anaesthesia care"[Title/Abstract] OR "post anesthesia care"[Title/Abstract] OR "perioperative"[Title/Abstract] OR "postanesthesia"[Title/Abstract] OR "postanaesthesia"[Title/Abstract] OR "intraoperative"[Title/Abstract] OR "intraoperative care"[Title/Abstract] OR "intraoperative care"[MeSH Terms]) AND ("emergency medical services"[MeSH Terms] OR "anesthesia department, hospital"[MeSH Terms] OR "anesthesia"[MeSH Terms] OR "Recovery Room"[MeSH Terms] OR "anesthesia"[Title/Abstract] OR "anaesthesia"[Title/Abstract] OR "PACU"[All Fields] OR "post anesthesia care unit*"[Title/Abstract] OR "post anaesthesia care unit*"[Title/Abstract] OR "recovery room*"[Title/Abstract] OR "emergency room*"[Title/Abstract] OR "anesthesia department*"[Title/Abstract] OR "anaesthesia department*"[Title/Abstract] OR "operating rooms/nursing"[MeSH Terms] OR "Operating Rooms"[MeSH Terms] OR "hospital*"[Title/Abstract] OR "post operative period"[Title/Abstract] OR "Hospitals"[MeSH Terms] OR "anesthesiology"[Title/Abstract] OR "anaesthesiology"[Title/Abstract] OR "Anesthesiology"[MeSH Terms])) AND ((fha[Filter]) AND (english[Filter] OR german[Filter]) AND (2007:2022[pdat]))
