# Supplementary material for: International competencies of nurses with advanced practice in anesthesia nursing: An integrative review
Source: Int J Nurs Stud Adv. 2025 Mar 17;8:100319. doi: 10.1016/j.ijnsa.2025.100319 (PMC11984995; doi:10.1016/j.ijnsa.2025.100319)
Supplement: Supplementary file 2 [file mmc2.docx]

**Supplement 2: Critical Appraisal**

|  | **Score based on JBI Critical Appraisal Checklist for qualitative studies** | | | | | | | | | | **Overall appraisal** |
| --- | --- | --- | --- | --- | --- | --- | --- | --- | --- | --- | --- |
| **Author** | **1** | **2** | **3** | **4** | **5** | **6** | **7** | **8** | **9** | **10** |  |
| Abelsson et al., 2021 | Y | Y | Y | Y | Y | N | Y | Y | Y | Y | Include, 3a |
| Averlid and Høglund (2020). | Y | Y | Y | Y | Y | N | Y | Y | Y | Y | Include, 3b |
| Dahlberg et al., 2022b | U | Y | Y | Y | Y | Y | Y | Y | Y | Y | Include, 3a |
| Everson et al., 2021 | Y | Y | Y | Y | Y | Y | N | Y | U | Y | Include, 3b |
| Rönnberg et al., 2018 | U | Y | Y | Y | Y | Y | Y | Y | Y | Y | Include, 3a |
| Schreiber and MacDonald, 2010 | U | Y | Y | Y | Y | N | N | Y | Y | Y | Include, 3a |
| Sundqvist and Carlsson, 2014 | U | Y | Y | Y | Y | Y | Y | Y | Y | Y | Include, 3a |
| Sundqvist et al., 2018 | N | Y | Y | Y | Y | Y | Y | Y | Y | N | Include, 3b |
| Y = yes, N = no, U = unclear, NA = not applicable. | | | | | | | | | | | |

|  | **Score based on JBI Critical Appraisal Checklist for Texts and Opinion** | | | | | | **Overall appraisal** |
| --- | --- | --- | --- | --- | --- | --- | --- |
| **Author** | **1** | **2** | **3** | **4** | **5** | **6** |  |
| Chen et al., 2020 | Y | U | Y | Y | Y | Y | Include, 5c |
| Egger-Halbeis and Schubert, 2008 | Y | Y | Y | Y | Y | Y | Include, 5b |
| Federico, 2007 | Y | U | Y | Y | Y | Y | Include, 5b |
| Fynes et al., 2014 | Y | Y | Y | Y | Y | Y | Include, 5a |
| Ide et al., 2020 | Y | Y | Y | Y | Y | Y | Include, 5b |
| Matsusaki and Sakai, 2011 | Y | U | Y | Y | Y | Y | Include, 5b |
| Rollison et al., 2021 | Y | Y | Y | Y | Y | U | Include, 5b |
| Y = yes, N = no, U = unclear, NA = not applicable. | | | | | | | |

|  | **Score based on JBI Critical Appraisal Checklist for Prevalence Studies** | | | | | | | | | **Overall appraisal** |
| --- | --- | --- | --- | --- | --- | --- | --- | --- | --- | --- |
| **Author** | **1** | **2** | **3** | **4** | **5** | **6** | **7** | **8** | **9** |  |
| Callan et al., 2021 | Y | U | N | N | U | Y | Y | Y | N | Include, 3c |
| Dahlberg et al., 2022a | U | Y | U | U | Y | Y | Y | Y | N | Include, 3b |
| Dahlberg et al., 2021 | Y | Y | Y | Y | Y | Y | Y | Y | Y | Include, 3a |
| Halakou et al., 2017 | U | Y | Y | Y | Y | Y | Y | Y | NA | Include, 3b |
| Herion et al., 2019 | Y | Y | U | Y | Y | Y | Y | Y | Y | Include, 3b |
| Lauridsen et al., 2015 | Y | Y | Y | Y | Y | Y | Y | Y | Y | Include, 3b |
| Meeusen et al., 2010 | Y | Y | Y | U | Y | U | Y | U | Y | Include, 3c |
| Olin et al., 2022 | U | N | N | Y | U | Y | Y | Y | NA | Include, 3b |
| Rayborn et al., 2017 | Y | Y | Y | Y | Y | Y | Y | Y | Y | Include, 3b |
| Sanclemente-Dalmau et al., 2022 | Y | Y | U | Y | U | Y | Y | Y | U | Include, 3b |
| Y = yes, N = no, U = unclear, NA = not applicable   \|  \| **Score based on McGill University Mixed Methods Appraisal Tool** \| \| \| \| \| \| \| **Overall appraisal** \| \| --- \| --- \| --- \| --- \| --- \| --- \| --- \| --- \| --- \| \| **Author** \| **1** \| **2** \| **3** \| **4** \| **5** \| **6** \| **7** \| \| Neft et al., 2013 \| Y \| Y \| N \| CT \| N \| N \| N \| Include, 3c \| \| Y = yes, N = no, CT = cannot tell. \| \| \| \| \| \| \| \| \| | | | | | | | | | | |

Abelsson, A., Falk, P., Sundberg, B., Nygardh, A., 2021. Empowerment in the perioperative dialog. *Nurs Open,* 8**,** 96-103. <https://doi.org/10.1002/nop2.607>.

Averlid, G., Høglund, J. S., 2020. The operating room as a learning arena: Nurse anaesthetist and student nurse anaesthetist perceptions. *J Clin Nurs,* 29**,** 1673-1683. <https://doi.org/10.1111/jocn.15227>.

Callan, V., Eshkevari, L., Finder, S., Jeter, L., May, S., Schirle, L., Stulce, J., Hoyem, R. L., Everson, M., 2021. Impact of COVID-19 Pandemic on Certified Registered Nurse Anesthetist Practice. *Aana j,* 89**,** 334-340.

Chen, Q., Lan, X., Zhao, Z., Hu, S., Tan, F., Gui, P., Yao, S., 2020. Role of Anesthesia Nurses in the Treatment and Management of Patients With COVID-19. *J Perianesth Nurs,* 35**,** 453-456. <https://doi.org/10.1016/j.jopan.2020.05.007>.

Dahlberg, K., Brady, J. M., Jaensson, M., Nilsson, U., Odom-Forren, J., 2021. Education, Competence, and Role of the Nurse Working in the PACU: An International Survey. *J Perianesth Nurs,* 36**,** 224-231 e6. <https://doi.org/10.1016/j.jopan.2020.08.002>.

Dahlberg, K., Jaensson, M., Flodberg, M., Mansson, S., Nilsson, U., 2022a. Levels of education and technical skills in registered nurses working in post-anaesthesia care units in Sweden. *Scand J Caring Sci,* 36**,** 71-80. <https://doi.org/10.1111/scs.12964>.

Dahlberg, K., Sundqvist, A. S., Nilsson, U., Jaensson, M., 2022b. Nurse competence in the post-anaesthesia care unit in Sweden: a qualitative study of the nurse's perspective. *BMC Nurs,* 21**,** 14. <https://doi.org/10.1186/s12912-021-00792-z>.

Egger Halbeis, C. B., Schubert, A., 2008. Staffing the operating room suite: perspectives from Europe and North America on the role of different anesthesia personnel. *Anesthesiol Clin,* 26**,** 637-63, vi. <https://doi.org/10.1016/j.anclin.2008.07.002>.

Everson, M., Wilbanks, B. A., Hranchook, A. M., Hirsch, M., Clayton, B. A., Jordan, L. M., Callan, V., 2021. From the Operating Room to the Front Lines: Shared Experiences of Nurse Anesthetists During the Coronavirus Pandemic. *Aana j,* 89**,** 109-116.

Federico, A., 2007. Innovations in care: the nurse practitioner in the PACU. *J Perianesth Nurs,* 22**,** 235-42. <https://doi.org/10.1016/j.jopan.2007.05.008>.

Fynes, E., Martin, D. S., Hoy, L., Cousley, A., 2014. Anaesthetic nurse specialist role: leading and facilitation in clinical practice. *J Perioper Pract,* 24**,** 97-102. <https://doi.org/10.1177/175045891402400502>.

Halakou, S., Bakhsha, F., Jafari, S. Y., Yousefi, Z., Aryaee, M., Yousefi, Mohammad Reza. 2017. The Clinical Competencies of Nurse Anesthetists in Response to Community Needs: A Delphi Study. *Journal of Clinical and Basic Research,* 1**,** 13-19. <https://doi.org/10.29252/jcbr.1.4.13>.

Herion, C., Egger, L., Greif, R., Violato, C., 2019. Validating international CanMEDS-based standards defining education and safe practice of nurse anesthetists. *Int Nurs Rev,* 66**,** 404-415. <https://doi.org/10.1111/inr.12503>.

Ide, Y., Umeno, Y., Tanaka, N., Nagamine, Y., Goto, T., Mcmullan, S. P., 2020. Introduction of evolving roles of Japanese perianesthesia nurses. *J Anesth,* 34**,** 719-722. <https://doi.org/10.1007/s00540-020-02826-3>.

Lauridsen, K. G., Schmidt, A. S., Adelborg, K., Lofgren, B., 2015. Organisation of in-hospital cardiac arrest teams - a nationwide study. *Resuscitation,* 89**,** 123-8. <https://doi.org/10.1016/j.resuscitation.2015.01.014>.

Matsusaki, T., Sakai, T., 2011. The role of Certified Registered Nurse Anesthetists in the United States. *J Anesth,* 25**,** 734-40. <https://doi.org/10.1007/s00540-011-1193-5>.

Meeusen, V., Van Zundert, A., Hoekman, J., Kumar, C., Rawal, N., Knape, H., 2010. Composition of the anaesthesia team: a European survey. *Eur J Anaesthesiol,* 27**,** 773-9. <https://doi.org/10.1097/EJA.0b013e32833d925b>.

Neft, M., Okechukwu, K., Grant, P., Reede, L., 2013. The revised scope of nurse anesthesia practice embodies the broad continuum of nurse anesthesia services. *Aana j,* 81**,** 347-50.

Olin, K., Göras, C., Nilsson, U., Unbeck, M., Ehrenberg, A., Pukk-Härenstam, K., Ekstedt, M., 2022. Mapping registered nurse anaesthetists' intraoperative work: tasks, multitasking, interruptions and their causes, and interactions: a prospective observational study. *BMJ Open,* 12**,** e052283. <https://doi.org/10.1136/bmjopen-2021-052283>.

Rayborn, M., Jeong, G., Hayden, S., Park, S., 2017. The Future of Certified Registered Nurse Anesthetist Practice in South Korea: Fading Into the Sunset or Breaking of a New Dawn? *Aana j,* 85**,** 361-368.

Rollison, S., Horvath, C., Gardner, B., Mcauliffe, M., Benson, A., 2021. Redeployment of Certified Registered Nurse Anesthetists During the Coronavirus Disease 2019 Pandemic. *Aana j,* 89**,** 133-140.

Rönnberg, L., Nilsson, U., Hellzen, O., Melin-Johansson, C., 2019. The Art Is to Extubate, Not to Intubate-Swedish Registered Nurse Anesthetists' Experiences of the Process of Extubation After General Anesthesia. *J Perianesth Nurs,* 34**,** 789-800. <https://doi.org/10.1016/j.jopan.2018.11.007>.

Sanclemente-Dalmau, M., Galbany-Estragues, P., Palomar-Aumatell, X., Rubinat-Arnaldo, E., 2022. Defining competencies for nurse anaesthetists: A Delphi study. *J Adv Nurs,* 78**,** 3696-3709. <https://doi.org/10.1111/jan.15348>.

Schreiber, R., MacDonald, M., 2010. Keeping Vigil over the Patient: a grounded theory of nurse anaesthesia practice. *J Adv Nurs,* 66**,** 552-61. <https://doi.org/10.1111/j.1365-2648.2009.05207.x>.

Sundqvist, A. S., Carlsson, A. A., 2014. Holding the patient's life in my hands: Swedish registered nurse anaesthetists' perspective of advocacy. *Scand J Caring Sci,* 28**,** 281-8. <https://doi.org/10.1111/scs.12057>.

Sundqvist, A. S., Nilsson, U., Holmefur, M., Anderzen-Carlsson, A., 2018. Promoting person-centred care in the perioperative setting through patient advocacy: An observational study. *J Clin Nurs,* 27**,** 2403-2415. <https://doi.org/10.1111/jocn.14181>.
